# Genome-wide survey of the *GATA* gene family in camptothecin-producing plant *Ophiorrhiza pumila*

**DOI:** 10.1186/s12864-022-08484-x

**Published:** 2022-04-03

**Authors:** Min Shi, Qikai Huang, Yao Wang, Can Wang, Ruiyan Zhu, Siwei Zhang, Guoyin Kai

**Affiliations:** Laboratory for Core Technology of TCM Quality Improvement and Transformation, School of Pharmaceutical Sciences, The Third Affiliated Hospital, Zhejiang Chinese Medical University, Hangzhou, Zhejiang 310053 P.R. China

**Keywords:** *Ophiorrhiza pumila*, GATA, Genome-wide, Camptothecin, Expression pattern

## Abstract

**Background:**

*Ophiorrhiza pumila* (Rubiaceae) is capable of producing camptothecin (CPT), one monoterpene indole alkaloid extensively employed in the treatment of multiple cancers. Transcription factors (TFs) *GATA* are a group of transcription regulators involved in plant development and metabolism, and show the feature of binding to the GATA motif within the promoters of target genes. However, *GATA* TFs have not been characterized in *O. pumila*.

**Result:**

In this study, a total of 18 *GATA* genes classified into four subfamilies were identified, which randomly distributed on 11 chromosomes of *O. pumila*. Synteny analysis of GATA genes between *O. pumila* and other plant species such as *Arabidopsis thaliana*, *Oryza sativa*, *Glycine max*, *Solanum lycopersicum*, *Vitis vinifera*, and *Catharanthus roseus* genomes were analyzed. Tissue expression pattern revealed that *OpGATA1* and *OpGATA18* were found to be correlated with *ASA*, *MK*, *CPR* and *GPPS,* which were highly expressed in leaves. *OpGATA7*, showed high expression in roots as most of the CPT biosynthetic pathway genes did, suggesting that these *OpGATAs* may be potential candidates regulating CPT biosynthesis in *O. pumila*.

**Conclusions:**

In this study, we systematically analyzed the OpGATA TFs, and provided insights into the involvement of OpGATA TFs from *O. pumila* in CPT biosynthesis.

**Supplementary Information:**

The online version contains supplementary material available at 10.1186/s12864-022-08484-x.

## Background

*Ophiorrhiza pumila* is a dicotyledonous plant classified into Rubiaceae family and remains as a sustainable source of camptothecin (CPT). CPT is a type of monoterpene indole alkaloids (MIAs) commonly used in treatment of cancers and was initially isolated from *Camptotheca acuminate* [1], and subsequently detected in *Nothapodytes nimmoniana* and other plants [2–4]. CPT inhibits tumor growth by blocking DNA topoisomerase I [5, 6]. Topotecan and irinotecan, two drugs developed by CPT derivatives, have been extensively employed in various cancers including lung, colorectal, cervical, and ovarian cancers [7]. The biosynthesis pathway of CPT is complex and remains not fully resolved [8]. Briefly, the terpene section of CPT is derived from the 2-C-methyl-d-erythritol 4-phosphate (MEP) and mevalonate (MVA) pathways. The produced geraniol is hydroxylated to 10-hydroxygeraniol under the catalysis of geraniol 10-hydroxy (G10H) [9–11], and then oxidized to 10-oxogeranial by 10-hydroxy geraniol oxidoreductase (10-HGO). Next, 10-oxogeraniol is converted to iridodial under the action of iridodial synthase, followed by conversion to iriotrial under the action of iridodial oxidoreductase (IO). Iriotrialis then converted to 7-deoxyloganetic acid under the action of IO. 7-deoxyloganetic acid is converted to 7-deoxyloganic acid by glucosyltransferase (7-DLGT) [6], and the product is then converted to loganic acid by 7-deoxyloganic acid hydroxylase (7-DLH) [6]. Lastly, secologanin, a precursor of CPT, is synthesized by secologanin synthase (SLS) [12, 13]. Another precursor tryptamine was produced from tryptophan under the catalyzation of tryptophan decarboxylase (TDC) [14]. Strictosidine is synthesized by condensation of tryptamine and secologanin catalyzed by strictosidine synthase (STR) [9, 15], and CPT is then formed via a series of catalytic reactions that have not yet been elucidated. A high-quality *O. pumila* genome has been assembled using next-generation sequencing, which led to a final genome assembly of 439.90 Mb, with contig and scaffold N50 values of 18.49 and 40.06 Mb, respectively. A total of 11 chromosomes were sequenced with sequential scaffolding strategy. Besides, more than 270 nitrogen-containing metabolites including different MIAs have been found [11]. Therefore, *O. pumila* has been regarded as a model plant for MIA biosynthesis [10].

To adapt to changes in the external environment and resist various biotic and abiotic stresses, plants have formed a series of complex and efficient regulatory networks causing changes in gene expression response to such stresses at multiple levels, and transcription factors (TFs) are critical regulators of these processes [16]. The GATA TF is one of the ubiquitous TF families in eukaryotes and is essential for many aspects of plant development, metabolism and signal conduction [17]. GATA proteins share a common feature of binding to the specific sequence (T/A) GATA (A/G) [18]. The DNA-binding domain of GATA contains a class IV zinc finger structure (C-X_2_-C-X_17–20_-C-X_2_-C), followed by a basal region [19]. Most *GATA* TFs in plants include a single C-X_2_-C-X_18_-C-X_2_-C motif and several contain C-X_2_-C-X_20_-C-X_2_-C. The first plant *GATA* TF was identified from tobacco and termed as *NTL1* harboring C-X_2_-C-X_18_-C-X_2_-C motif [20]. GATA TFs have been identified and characterized in *A. thaliana* (29), and *O. sativa* (28) [17]. Based on phylogenetic relationships, DNA binding regions, and intron-exon structures, *Arabidopsis* and rice GATA family genes can be categorized into four families including I, II, III, and IV [21].

GATA TFs have been reported involved in plant metabolism. For example, *A. thaliana* GATA nitrate-inducible carbon-metabolism-involved (GNC) and cytokinin-responsive GATA1 (CGA1) regulated chlorophyll levels, chloroplast size, photosynthetic efficiency, and carbon and nitrogen metabolism [22]. Moreover, *GNC* and *CGA1* show high expression in green tissues and are capable of mediating cytokinin to regulate plastid development [23]. *GATA8* mediated the biomass accumulation and photosynthetic efficiency in *O. sativa* seedlings [24]. Transient overexpression of *CrGATA1* in *Catharanthus roseus* seedlings increased vindoline production by activating D4H gene which contained GATA motifs in the promoter [25]. Under low nitrogen deposition, *GATA44* and *GATA58* genes exhibit low expression in soybean seedlings [26]. Additionally, in higher plants, the assimilation pathway of nitrate is tightly regulated. Nitrate is reduced from nitrate reductase to nitrite and then to ammonium nitrogen (NH4^+^) by nitrite reductase to participate in the synthesis of amino acids and proteins [27]. The promoter of nitrate reductase (NIA) in tomatoes covers the required *cis*-acting regulatory elements capable of specifically recognizing and binding to GATA protein and then regulating nitrogen metabolism [28]. Moreover, ammonia is required for alkaloid biosynthesis. Nitrogen is an important nutritional factor affecting plant alkaloid biosynthesis and accumulation [29], and CPT contains two nitrogen atoms in its molecular structure owing to its origin as an amino acid-derived alkaloid [11]. Thus, nitrogen metabolism may critically affect the regulation of CPT.

By analyzing the promoter sequence of CPT biosynthetic genes, multiple GATA motifs were deserved, suggesting exploring GATA TFs from *O. pumila* (*OpGATA*s) is of importance to study CPT biosynthesis. In this study, GATA family TFs distributed in *O. pumila* was systematically characterized. Based on phylogenetic relationship and expression pattern combined with co-expression analysis, candidate *GATA* genes regulating CPT biosynthesis were predicted. The results provided a comprehensive analysis of *OpGATA* family genes, which shed new lights on CPT biosynthesis in *O. pumila*.

## Results

### Identification and phylogenetic analysis of GATA proteins

In this study, a total of 18 *OpGATA* genes were identified from the genome of *O. pumila* according to HMM search results, and renamed as *OpGATA1-OpGATA18* according to their chromosome position. Fundamental characteristics of *OpGATA1-OpGATA18* including coding sequence length, protein molecular weight, point isoelectric (pI) and subcellular location were analyzed (Table S1). The complete open reading frame (ORF) of OpGATAs varied from 160 bp (OpGATA4) to 543 bp (OpGATA15), and the molecular weights ranged from 17.73 kDa to 59.86 kDa. The pI values were predicted ranging from 5.05 (OpGATA4) to 10.11 (OpGATA6). All OpGATA proteins were predicted to localize in the nucleus (Table S1).

### Phylogenetic analysis and classification of OpGATA

**Fig. 1:**
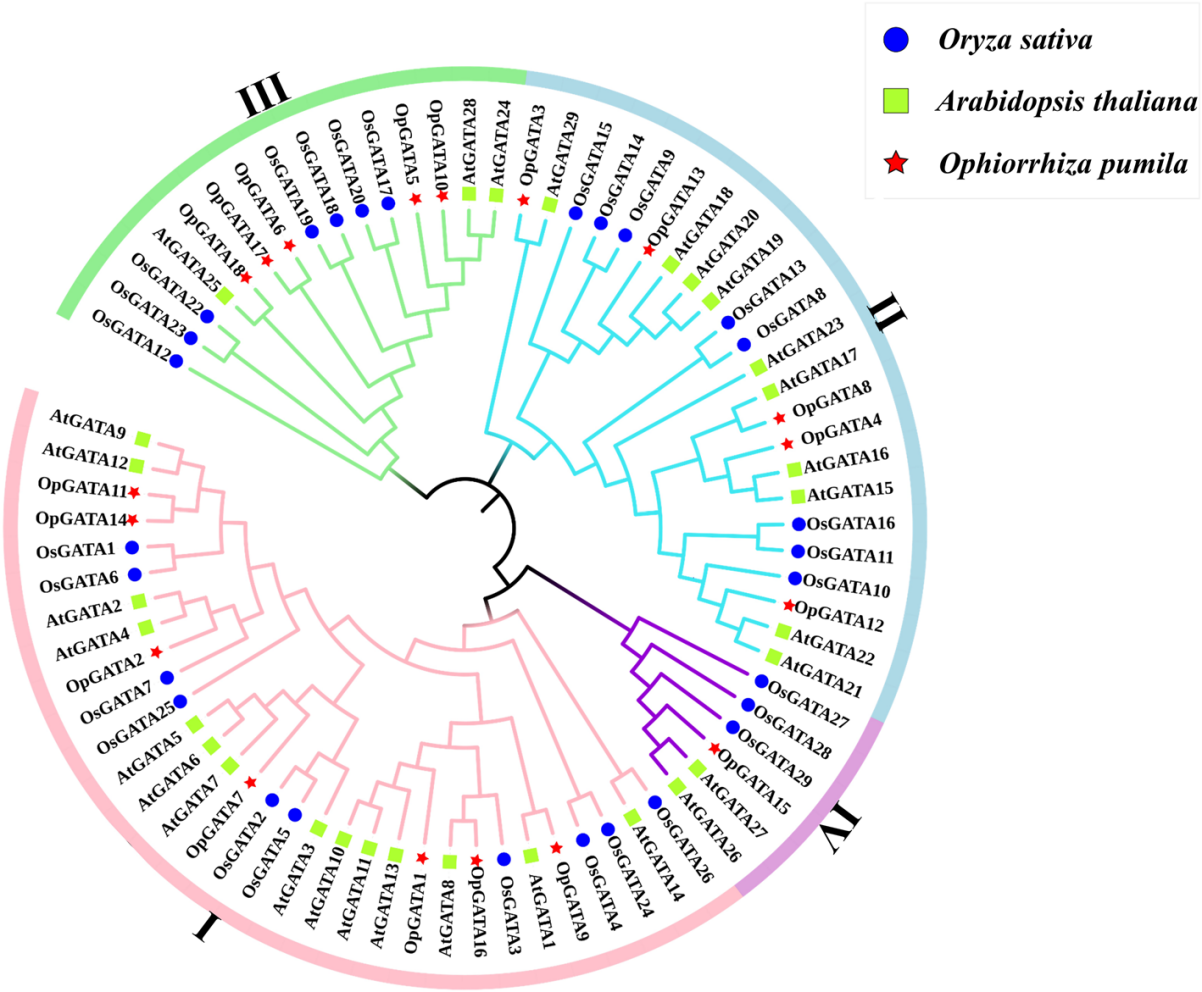
Neighbor-joining tree representing the relationship among GATA proteins of *O. pumila*, *O. sativa* and *A. thaliana*. Constructed with MEGA v7 using full-length amino acid sequences and the bootstrap test replicate was set as 1000 times

**Fig. 2:**
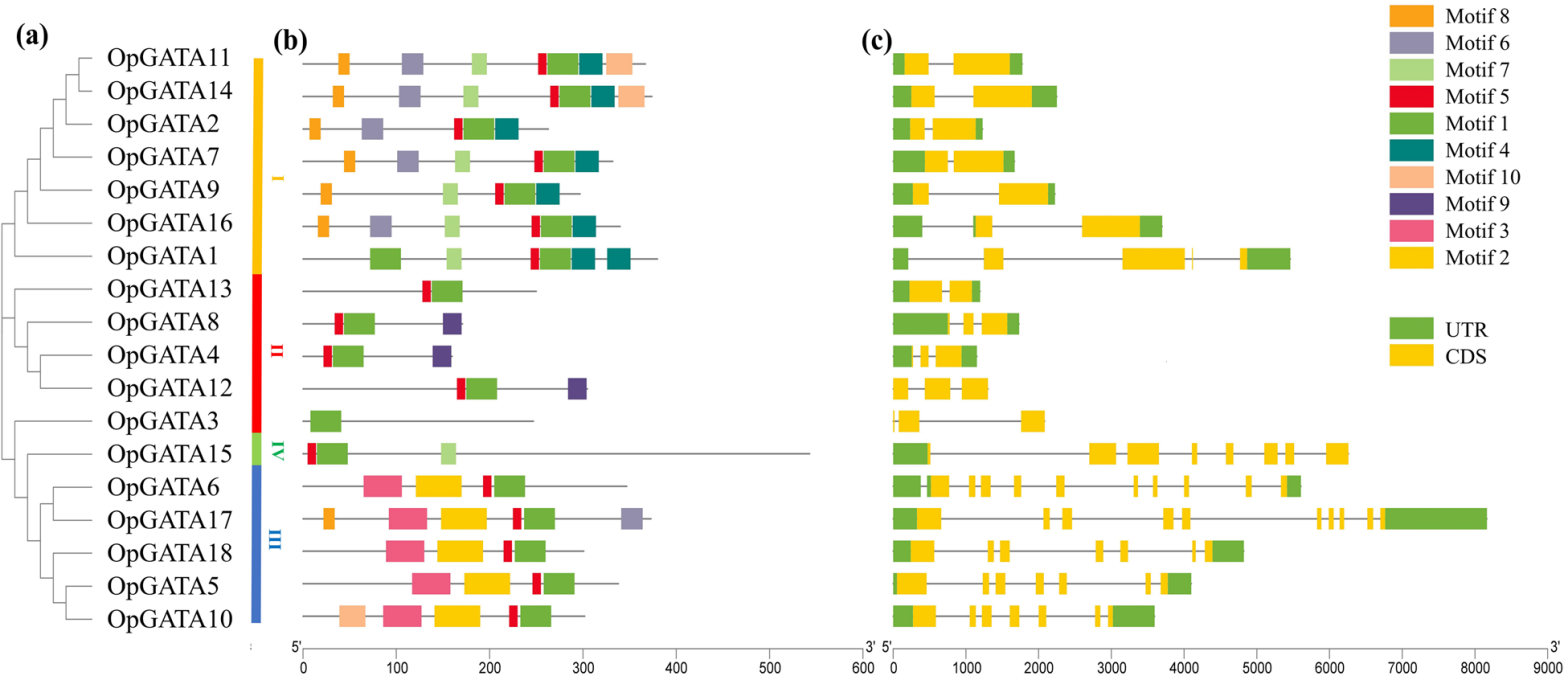
Schematic representation of phylogenetic relationships, conserved motifs and gene structures of the GATA genes in *O. pumila*. **a** A phylogenetic tree of 18 OpGATA proteins. **b** The motif composition of OpGATA proteins. The motifs are displayed in different colored boxes. **c** Exon/intron structures of *OpGATA* genes

To determine the phylogenetic relationships of OpGATA proteins, a neighbor-joining tree was constructed by complying with the full-length GATA proteins from *O. pumila*, *O. sativa*, *A. thaliana* and *C. roseus* (Fig. 1; Fig. S1). OpGATA proteins were classified into four distinct subfamilies (I, II, III, and IV) (Fig. 2a). Seven OpGATAs (OpGATA1, OpGATA2, OpGATA7, OpGATA9, OpGATA11, OpGATA14, and OpGATA16) were classified as subfamily I; five OpGATAs (OpGATA3, OpGATA4, OpGATA8, OpGATA12, and OpGATA13) were assigned into subfamily II; five OpGATAs (OpGATA5, OpGATA6, OpGATA10, OpGATA17, and OpGATA18) were grouped into subfamily III and subfamily IV having only one OpGATA namely OpGATA15.

### Gene structure and motif composition of the OpGATA gene family

**Fig. 3:**
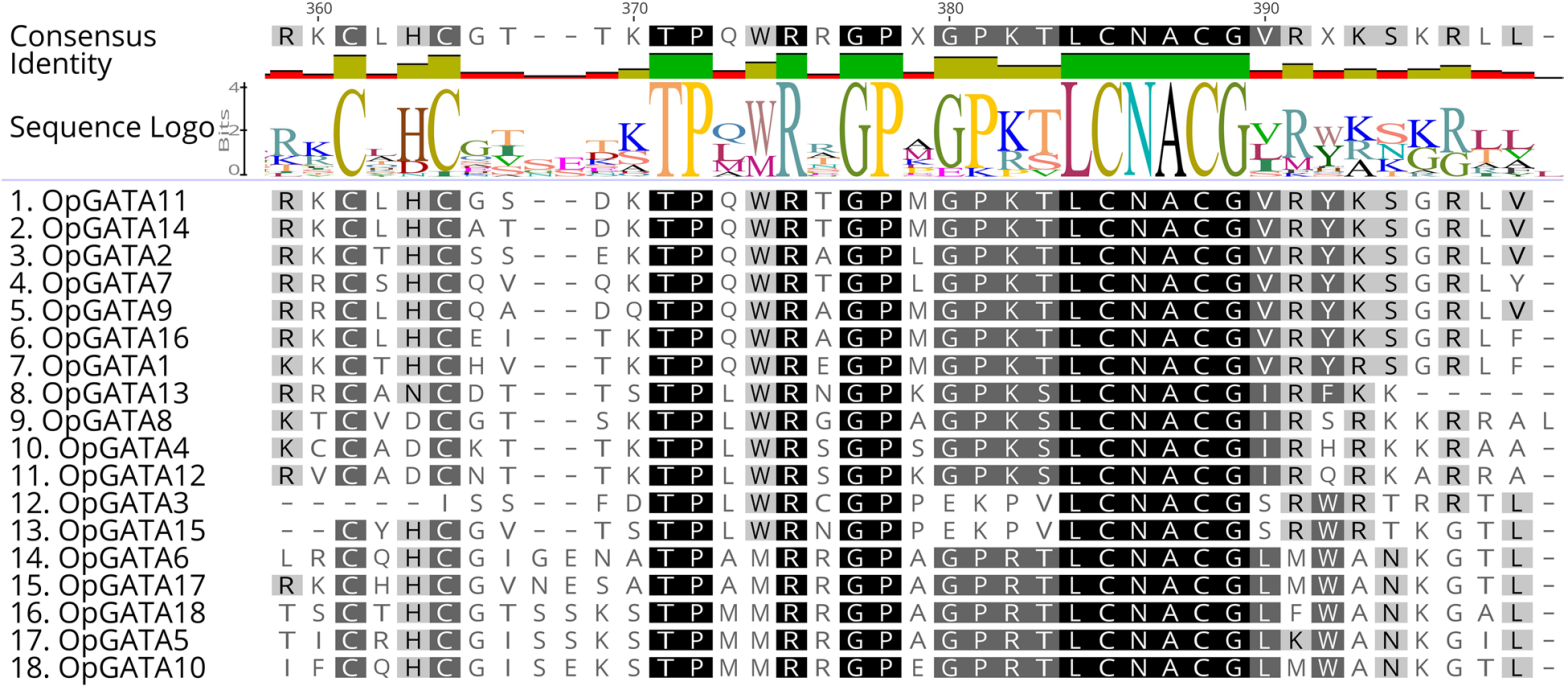
Alignments of GATA domain sequences of the GATA family members in *O. pumila*

To gain insights into the characteristics of OpGATA proteins, the motifs of OpGATA proteins were analyzed by MEME. In total, 10 different conservative motifs were characterized (motifs 1–10) (Fig. 2b, Table S2). Motifs 1 and 5 were detected in all proteins except OpGATA3. Motifs 4, 6, 7, and 8 were mainly observed in subfamily I; motif 9 was mainly present in subfamily II; motif 2 and 3 remained in subfamily III; and motifs 1, 5, and 7 were mainly contained in subfamily IV. The exon and intron structures of *OpGATA* genes were obtained by comparing the corresponding genomic DNA sequences of *O. pumila*. Notably, subfamilies III and IV had more introns, whereas subfamilies I and II had only 1–3 introns (Fig. 2c). Overall, members within a single subfamily exhibited similar gene structures, and the results in this study showed similar gene structures and conserved motifs, strongly supporting the results of phylogenetic analysis of subfamily classification. Similar to previous studies of *A. thaliana*, rice, and other plants [17, 19, 21], OpGATAs classified into subfamilies I, II, and IV contain the conserved domain C-X_2_-C-X_18_-C-X_2_-C (except OpGATA3 and OpGATA15), while domain with the C-X_2_-C-X_20_-C-X_2_-C structure was existed in subfamily III (Fig. 3).

### Chromosomal distribution and synteny analysis of the OpGATA gene family

**Fig. 4:**
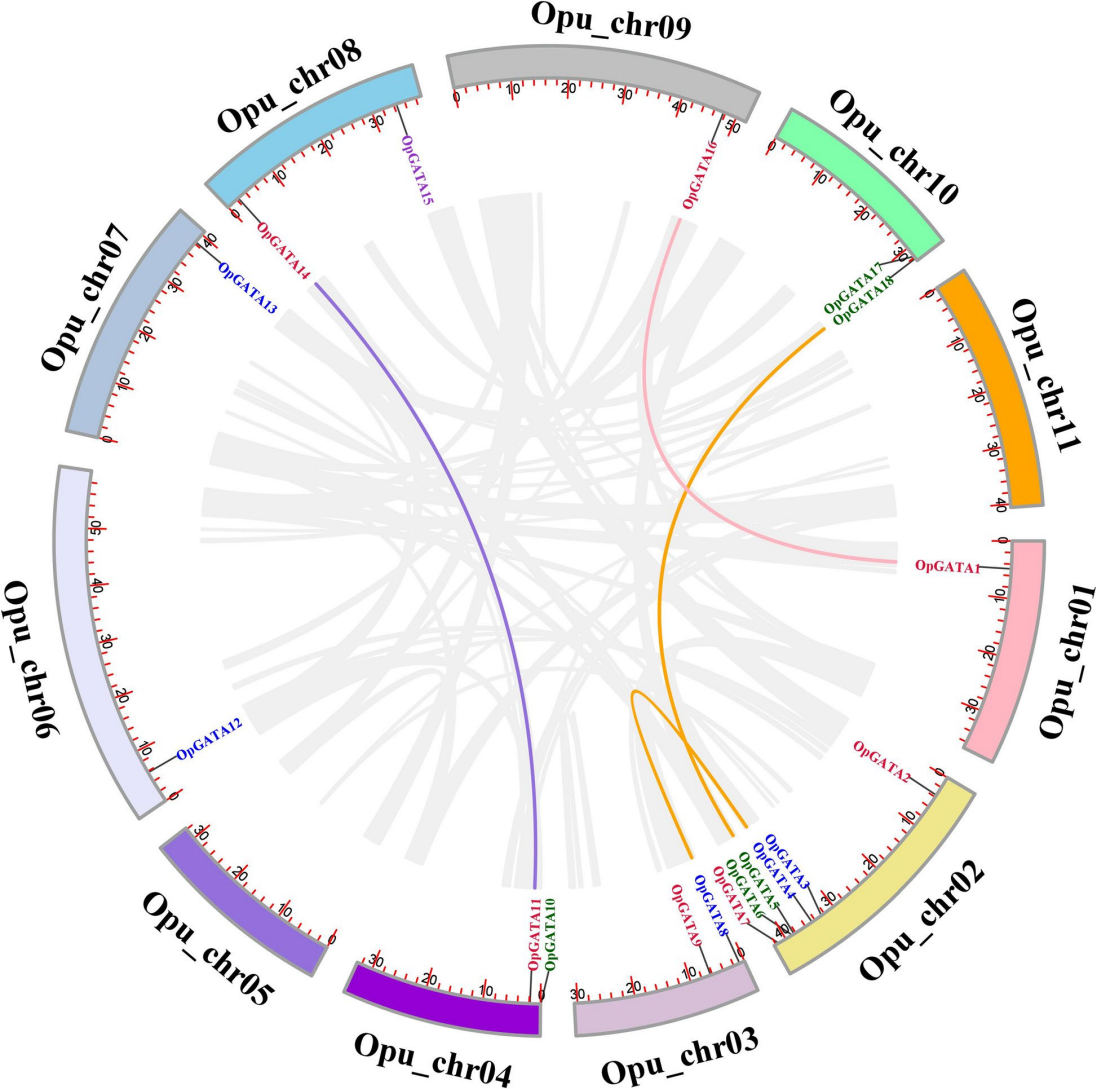
The chromosomal distribution and synteny analysis of *OpGATA* genes in *O. pumila.* The locations of all the *OpGATA* genes are depicted in the chromosomes. Red-colored genes belong to subfamily I, blue-colored genes belong to subfamily II, green-colored genes belong to subfamily III, purple-colored genes belong to subfamily IV. Background gray lines indicate all *O. pumila* genome synteny blocks, and the Colored lines highlight the duplicated *OpGATA* gene pairs. ID of the chromosomes is indicated at the bottom

A physical location map of all *OpGATA* genes in the genome of *O. pumila* was drawn (Fig. 4). The distribution of *OpGATA* genes on chromosomes was not homogeneous. The maximum number of *OpGATA* genes was distributed on Opu_Chr02 (OpGATA2-OpGATA7), whereas Opu_chr05 and Opu_chr11 had no *OpGATA* genes and the other chromosomes harbored 1–2 *OPGATA* genes, such as OpGATA1 distributed on Opu_Chr01, OpGATA8 and OpGATA9 on Opu_Chr03, OpGATA10 and OpGATA11 on Opu_Chr04, OpGATA12 on Opu_Chr06, OpGATA13 on Opu_Chr07, OpGATA14 and OpGATA15 on Opu_Chr08, OpGATA16 on Opu_Chr09, OpGATA17 and OpGATA18 on Opu_Chr10.

Moreover, replication events of OpGATAs were analyzed. The result showed that no tandem repeats were identified among the 18 genes, while four pairs of fragment repeats were detected between eight chromosomes, which were Opu_chr01 (*OpGATA1*)/Opu_chr09 (*OpGATA16*), Opu_chr02 (*OpGATA4*)/Opu_chr03 (*OpGATA8*), Opu_chr02 (*OpGATA5*)/Opu_chr10 (*OpGATA18*), and Opu_chr04 (*OpGATA11*)/Opu_chr08 (*OpGATA14*). Accordingly, some *OpGATA* genes may have been generated by gene replication, thereby critically affecting the amplification of *OpGATA* genes in *O. pumila*.

**Fig. 5:**
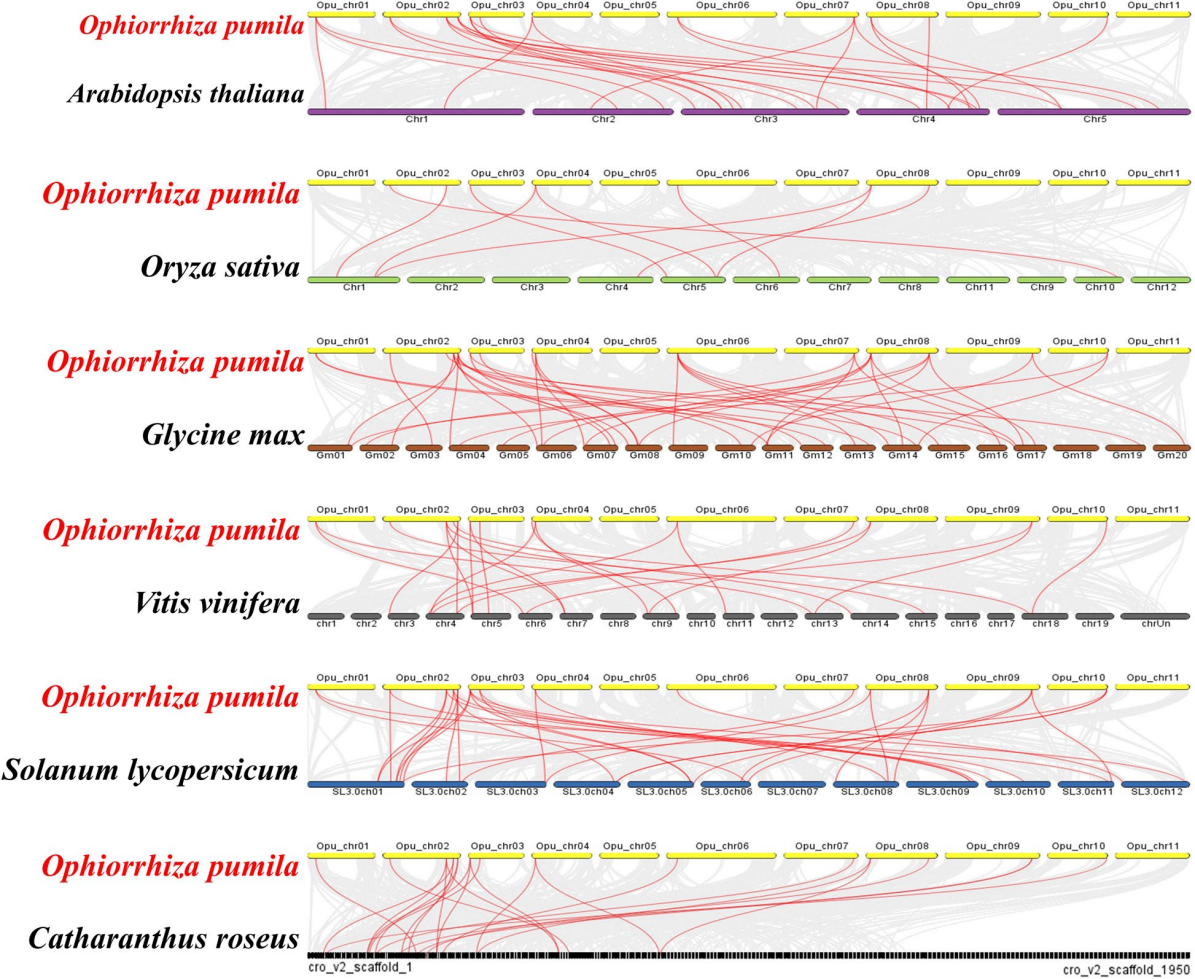
Synteny analysis of GATA genes between *O. pumila* and five representative plant species (*A. thaliana*, *O. sativa*, *G. max*, *S. lycopersicum*, *V. vinifera* and *C. roseus*). Gray lines in the background indicate the collinear blocks within *O. pumila* and other plant genomes, while red lines highlight syntenic GATA gene pairs

To gain insights into the evolution of *O. pumila* GATA family, represented comparative system diagrams comparing *O. pumila* and five other dicotyledonous plants (*A. thaliana*, *G. max*, *S. lycopersicum*, *V. vinifera* and *C. roseus*) and one monocotyledonous plant (*O. sativa*) was analyzed (Fig. 5, Table S3). In total, *OpGATA* genes displayed different syntenic relationship with *G. max* (41), *S. lycopersicum* (35), *V. vinifera* (23), *A. thaliana* (26), *C. roseus* (23) and *O. sativa* (9), respectively, suggesting that GATA genes from *O. pumila* relatively had a more similar relationship with that in soybean. Furthermore, OpGATA4, OpGATA8, OpGATA12, and OpGATA14 exhibited syntenic relationship in the six plant species, demonstrating that these four proteins may critically affect evolution.

### Expression profiles of OpGATA genes and key enzyme genes in different samples

**Fig. 6:**
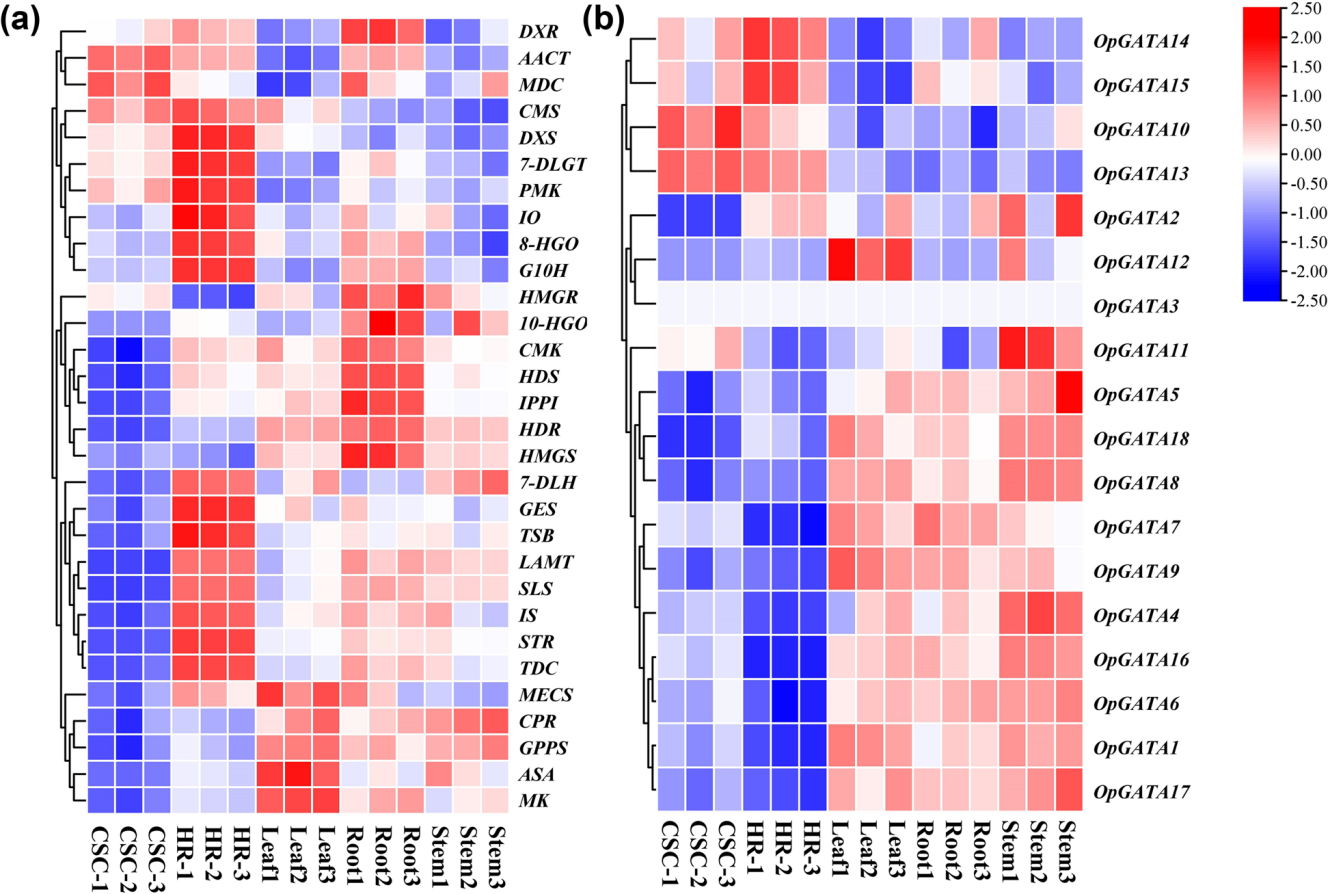
The expression patterns of key enzyme genes and OpGATAs in leaves, roots, stems, cell suspension cultures and hairy roots examined by QRT-PCR. The color scale represents relative expression levels from high (red color) to low (blue color)

**Fig. 7:**
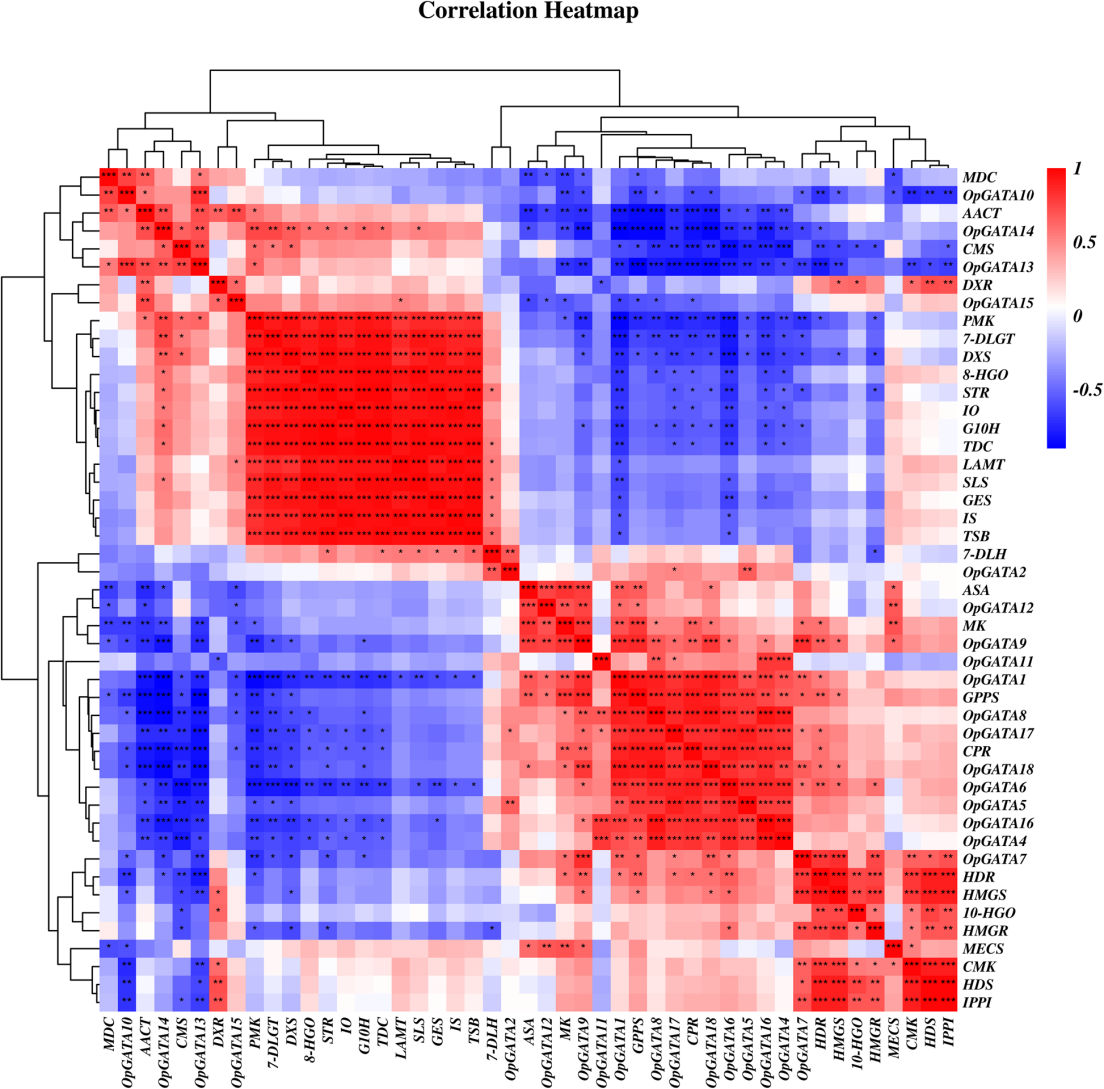
The correlation between the gene expression patterns of OpGATA and key enzyme genes. Red: positively correlated; blue: negatively correlated. Significant difference was calculated, *P < 0.05, **P < 0.01, ***P < 0.001

Expression profiles of *OpGATA* and vital enzyme genes were evaluated in three distinct tissues and organs (roots, stems and leaves), together with cell suspension cultures and hairy roots. A heat map was built according to the QRT-PCR analysis. The results showed that most of the CPT-producing pathway genes (*CMS*, *DXS*, *7-DLGT*, *PMK*, *IO*, *8-HGO*, *G10H*, *HMGR*, *10-HGO*, *CMK*, *HDS*, *IPPI*, *HDR*, *HMGS*, *7-DLH*, *GES*, *TSB*, *LAMT*, *SLS*, *IS*, *STR* and *TDC*) expressed highly in roots or hairy roots (Fig. 6a). Several genes including *MECS*, *CPR*, *MK*, *ASA*, and *GPPS* showed higher expression in leaves. As indicated in Fig. 6b, most of *OpGATA* genes were highly expressed in stems, while expression level of *OpGATA9* and *OpGATA12* was higher in leaves, and *OpGATA7*, *OpGATA14* as well as *OpGATA15* expressed higher in roots. In addition, correlations between *OpGATA*s and pathway genes were analyzed (Fig. 7, Table S4). The results showed that *OpGATA7* exhibited positive associations with key enzyme genes showing high expression in roots. Among this, *HMGS* and *HDR* showed pole-strength correlations (*p* < 0.05, *r* > 0.8) with *OpGATA7*, along with *CMK*, *GPPS*, *HDS*, *HMGR* IPPS showed strong correlations (*p* < 0.05, 0.6 < *r* < 0.8). *OpGATA1*, *OpGATA4*, *OpGATA5*, *OpGATA6*, *OpGATA8*, *OpGATA9*, *OpGATA12*, *OpGATA16*, *OpGATA17*, and *OpGATA18* were found to be correlated with *ASA*, *MK*, *CPR*, or *GPPS* which were highly expressed in leaves. Only *OpGATA1* and *OpGATA18* exhibited a positive correlation with all the four genes, with Pearson correlation coefficients of greater than 0.6. To identify the *cis*-element of *OpGATA*, the 3000 bp promoter sequences of genes encoding vital enzymes in the CPT biosynthesis pathway were analyzed using PlantCARE (http://bioinformatics.psb.ugent.be/webtools/plantcare/html/). As predicted, the GATA motif was present in the promoters of several key enzyme genes (*TSB*, *SLS*, *MK*, *MDC*, *IPPI*, *HMGR*, *HDR*, *GES*, *G10H*, *DXS*, *AACT*, and *8-HGO*) (Table S5). Thus, these key biosynthetic genes may be regulated by *GATA* TFs.

## Discussion

Camptothecin (CPT) is a widely known monoterpene indole alkaloid with excellent anticancer activity. CPT has been isolated from different plant species. CPT- producing weedy plant *O. pumila* has brought about widespread attention, and the whole genome of *O. pumila* has been sequenced [29]. Recently, metabolic engineering has been applied in *O. pumila* to elevate CPT content. For example, individual introduction of *G10H* or *SLS*, and co-expression of *G10H* and *SLS* significantly enhanced CPT content in transgenic *O. pumila* hairy roots [30]. Besides, transcription regulation of CPT biosynthesis has been studied. RNA interference of *OpERF2* suppressed expression level of genes involved in MEP and secologanin-strictosidine pathways [31]. The transcription repressor OpMYB1 reduced CPT biosynthesis by downregulating expression level of *TDC* [32]. OpWRKY2 acted as a positive regulator of CPT biosynthesis by directly targeting *TDC* [10]. OpWRKY1 inhibited CPT biosynthesis by directly down-regulating CPR transcription in *O. pumila* [13]. Nevertheless, transcription regulation of CPT biosynthesis needs further study.

The GATA TF family involved in many aspects of physiology-related processes has been broadly explored in a range of plants including *Arabidopsis*, rice [17], grapes [33], *Moso bamboo* [34], and *Gossypium* sp. [21]. The present study reported GATA TFs in *O. pumila*. Totally, 18 GATA TFs were identified and named *OpGATA1-OpGATA18* according to their physical location on the chromosome. The whole OpGATA family in *O. pumila* could be classified into four groups, similar to those in *A. thaliana*. In subfamily III, the GATA domain harbored 20 residues in the zinc finger domain, making up a C-X_2_C-X_20_-C-X_2_-C structure, and the other three subfamilies showed that C-X_2_C-X_18_-C-X_2_-C structure, containing 18 residues. The CCT and TIFY domains were specifically identified within subfamily III. The CCT domain was initially found in *Arabidopsis* Constans protein, which facilitates root and hypocotyl development within *A. thaliana* and mediates flowering [35]. Previously, the family with a completely conserved TIFY domain was termed TIFY [36]. However, in recent studies, the TIFY domain has been shown to exist extensively in jasmonate ZIM domain protein family and PEAPOD proteins, which are associated with the jasmonic acid pathway [37].

Motif analysis showed that all OpGATAs contained motif 1 and 5 except OpGATA3, and specific motifs were detected in other groups. For example, motif 4 was only observed in subfamily I, motif 9 was only detected in subfamily II, and motifs 2 and 3 were only detected in subfamily III, suggesting that although some motifs of GATA family genes are highly conserved, new evolutionary motifs may have distinct functions in some plants, and the functions of these new evolutionary motifs need to be further verified. The homology of *GATA* genes from *O. pumila* with those from *Arabidopsis*, rice, soybeans, tomatoes, and grapes was also explored. Notably, the *Arabidopsis* GATA TFs AtGATA1 (AT3G24050), AtGATA2 (AT3G60530), and AtGATA4 (AT2G45050) have been reported to facilitate light-dependent regulation of gene expression and photomorphogenesis [38]. Accordingly, the homologous genes OpGATA9 (Opuchr03_g0010130–1.1) and OpGATA2 (Opuchr03_g0010130–1.1) may also affect light-dependent regulation of genes [38]. AtGATA22 (AT4G26150), which is homologous with OpGATA12 (Opuchr06_g0009000–1.1), affects the response to cytokinins and hinders root growth in *A. thaliana* [39]. Additionally, *GNC* (AT5G56860), which is homologous to OpGATA12, adversely affects seed germination, flowering, and leaf elongation, and overexpression of GNC inhibits the germination, leaf expansion, and flowering of *A. thaliana* [40]. AtGATA12 (AT5G25830), which is homologous to OpGATA14, is involved in primary dormancy in *A. thaliana* [41].

Expression level of most genes encoding the key enzymes in the CPT biosynthesis pathway (*CMS*, *DXS*, *7-DLGT*, *PMK*, *IO*, *8-HGO*, *G10H*, *HMGR*, *10-HGO*, *CMK*, *HDS*, *IPPI*, *HDR*, *HMGS*, *7-DLH*, *GES*, *TSB*, *LAMT*, *SLS*, *IS*, *STR* and *TDC*) were significantly higher in roots or hairy roots compared with those in other tissues, whereas genes involved in the MVA and MEP pathways (*ASA*, *GPPS*, *MK*, and *MECS*) were mostly expressed in leaves. *OpGATA7* exhibited positive associations with CPT biosynthesis pathway genes, which showed significant expression in roots. Plant terpenoids are synthesized mainly through the MVA and MEP pathways [42]. Genes that are highly expressed in the leaves are typically involved in the MEP and MVA pathways [43], demonstrating that *OpGATA1*, *OpGATA4*, *OpGATA5*, *OpGATA6*, *OpGATA8*, *OpGATA9*, *OpGATA12*, *OpGATA16*, *OpGATA17*, and *OpGATA18* may regulate CPT biosynthesis by participating in the upstream pathway. Among the genes mentioned above, GATA motifs were found in the promoters of key enzyme genes (i.e., *SLS*, *MDC*, *IPPI*, *HMGR*, *HDR*, *GES*, *G10H*, *AACT*, and *8-HGO*) highly expressed in roots or hairy roots and key enzyme genes (i.e., *MK*, *DXS*) highly expressed in leaves, demonstrating that these key enzyme genes may be directly regulated by *OpGATA* genes and then affect the biosynthesis of CPT. Of which, the genes highly expressed in roots or hairy roots which is pole-strength correlations with *OpGATA7* is a higher possibility of being directly regulated. It is interesting that OpGATA3 expressed ubiquitously in the stem, roots, leaves, cell suspension cultures and hairy roots, we analyzed and found that the promoter of OpGATA3 has phytohormone responsive elements and biotic and abiotic stress (Fig. S2), which is in close relation to the regulation of plant growth.

Overall, this comprehensive analysis of *GATA* family genes from *O. pumila* provided insights into the characteristics of *OpGATA* genes and may improve our understanding of the mechanisms regulating CPT biosynthesis in *O. pumila*.

## Conclusion

In this study, OpGATA TF family in *O. pumila* were characterized and identified. Overall, a total of 18 *OpGATA* genes showing different chromosomal distribution were classified into four subfamilies. Synteny analysis of GATA genes were conducted within several plant species including *O. pumila*, *Arabidopsis*, grapes, tomatoes, soybeans and *C. roseus*, and the functions of some homologous genes were predicted. OpGATA genes showed different expression patterns within a range of samples (leaves, stems, roots, cell suspension cultures and hairy roots) in correlation to key pathway genes, highlighting the potential roles of some OpGATA genes in the regulation of CPT biosynthesis in *O. pumila*. This study provides novel OpGATA TFs involved in regulating CPT biosynthesis.

## Materials and methods

### Identification of OpGATAs

The hidden Markov model (PF00320) of the GATA domain originating from the Pfam database (http://pfam.xfam.org) was used to identify the *OpGATA* family. To avoid probable GATA members were missing, a BLASTP-algorithm based search using GATA amino acid sequences from Arabidopsis as queries was conducted: e-value ≤1e^− 3^ [44]. The Pfam database (http://pfam.xfam.org/search/sequence), NCBI CDD (https://www.ncbi.nlm.nih.gov/Structure/cdd/wrpsb.cgi) and BLASTP (https://blast.ncbi.nlm.nih.gov/Blast.cgi) were employed to verify the integrity of the GATA domain, with an e-value cutoff of 0.01 [45]. The ProSite ExPASy server (http://web.expasy.org/protparam/) was adopted to predict the physical and chemical properties of OpGATA proteins. Subcellular localization of GATA proteins was predicted using CELLO (http://cello.life.nctu.edu.tw/).

### Multiple sequence alignment and phylogenetic analysis

The GATA proteins from *A. thaliana* Information Resources (www.arabidopsis.org/index.jsp) and the Rice Genome Annotation Project (http://rice.plantbiology.msu.edu/cgi-bin/ORF_infopage.cgi) were downloaded [19]. MAFFT software was employed for multiple sequence alignment of GATA proteins [46]. The neighbor-joining tree of GATA TF families from *A. thaliana*, *O. sativa*, *O. pumila* and *C. roseus* were built by MEGA v7 [47], with the parameters of Poisson model, pairwise deletion, and 1000 bootstrap tests.

### Motifs and gene structures

The MEME was employed (http://meme.sdsc.edu/meme/itro.html) to identify the conserved motif of GATA protein in *O. pumila*, with the following parameters were adopted: 0 or 1 occurrence per sequence; maximum number of motifs = 10; and optimum motif length = 6–50 residues. Exon-intron structure of the *GATA* members was investigated by analyzing the *O. pumila* genome, and gene structure was visualized with TBtools [48].

### Chromosomal distribution and gene duplication of GATA genes

The method for mapping *GATA* genes on the chromosome of *O. pumila* was identical to that of *FtAP2/ERF* genes [49]. Gene replication events were investigated using the multiple collinear scanning toolkit (MCScanX) and BLASTP method. TBtools software (https://github.com/CJ-Chen/TBtools) was adopted to build syntenic analysis maps for determining the syntenic relationships between OpGATA proteins and GATA proteins from *A. thaliana*, *O. sativa*, *G. max*, *S. lycopersicum*, *V. vinifera* and *C. roseus*.

### Expression analysis by quantitative real-time PCR (QRT-PCR)

Total RNA was extracted using a Plant RNAprep Pure Kit (TIANGEN, China). Corresponding sequences of *OpGATA* genes and key enzyme genes were acquired from the *O. pumila* genome sequence database (http://pumila.kazusa.or.jp/). Primers used for QRT-PCR analysis were designed using Primer 5 software (Table S6). Relative expression levels were calculated using the 2^−ΔΔCt^ method, with housekeeping gene *OpActin* from *O. pumila* as the internal control [10]. All QRT-PCR analyses were performed with three biological replicates. The heatmap was constructed by TBtools software base on QRT-PCR analysis. Pearson’s correlation coefficient was analyzed using the OmicStudio tools at https://www.omicstudio.cn, significant difference was tested at significance levels of 0.05, 0.01, and 0.001.

## Supplementary Information

**Additional file 1.**

**Additional file 2.**

**Additional file 3.**

**Additional file 4.**

**Additional file 5.**

**Additional file 6.**

**Additional file 7.**

## Data Availability

The genomic information of *Ophiorrhiza pumila* was downloaded from *Ophiorrhiza pumila* Genome DateBase (http://pumila.kazusa.or.jp/). All data generated or analyzed during this study are included in this published article and its supplementary information files.

## Abbreviations

*O. pumila*: *Ophiorrhiza pumila*
*A. thaliana*: *Arabidopsis thaliana*
*O. sativa*: *Oryza sativa*
*G. max*: *Glycine max*
*S. lycopersicum*: *Solanum lycopersicum*
*V. vinifera*: *Vitis vinifera*
CPT: Camptothecin
pI: Point isoelectric
TFs: Transcription factors
MIAs: Monoterpene indole alkaloid
MEP: 2-C-methyl-d-erythritol 4-phosphate
MVA: Mevalonate
G10H: Geraniol 10-hydroxy
10-HGO: 10-hydroxy geraniol oxidoreductase
IO: Iridodial oxidoreductase
7-DLGT: 7-deoxyloganic acid by glucosyltransferase
7-DLH: 7-deoxyloganic acid hydroxylase
SLS: Secologanin synthase
TDC: Tryptophan decarboxylase
STR: Strictosidine synthase
ASA: Anthranilate synthase
CPR: Cytochrome P450 reductases
DXR: 1-deoxy-D-xylulose-5-phosphate reductoisomerase
DXS: 1-deoxy-D-xylulose-5-phosphate synthase
HDR: 1-hydroxy-2-methyl-2(E)-butenyl-4-diphosphate reductase
TSB: The beta-subunit of tryptophan synthase
QRT-PCR: Quantitative real-time PCR
8-HGO: 8-hydroxy-geraniol oxidoreductase
AACT: Acetyl-CoA C-acetyltransferase
CMK: 4-(cytidine 5-diphospho)-2-C-methylerythritolkinase
CMS: 4-(cytidine 5-diphospho)-2-C-methylerythritol synthase
GES: Geraniol synthase
GPPS: Geranyl diphosphate synthase/geranyl pyrophosphate synthase
HDS: Hydroxymethylbutenyl 4-diphosphate synthase
HMGR: 3-hydroxy-3-methylglutaryl-CoA reductase
HMGS: 3-hydroxy-3-methylglutaryl-CoA synthase
IPPI: Isopentenyl diphosphate isomerase
IS: Iridoid synthase
LAMT: Loganic acid O-methyltransferase
MDC: Mevalonate 5-diphosphate decarboxylase/mevalonate (diphospho)-decarboxylase
MECS: 2-C-methylerythritol-2,4-cyclodiphosphate synthase
MK: Mevalonate kinase
PMK: Phosphomevalonate kinase
CSC: Cell suspension cultures
HR: Hairy roots
