# Supplementary material for: Genome-wide survey of the GATA gene family in camptothecin-producing plant Ophiorrhiza pumila
Source: BMC Genomics. 2022 Apr 3;23:256. doi: 10.1186/s12864-022-08484-x (PMC8977026; doi:10.1186/s12864-022-08484-x)

Figure S1 Neighbor-joining tree representing the relationship of GATA proteins among *O. pumila*, *O. sativa*, *A. thaliana* and *C. roseus*. Constructed with MEGA7 using full-length amino acid sequences and the bootstrap test replicate was set as 1000 times.


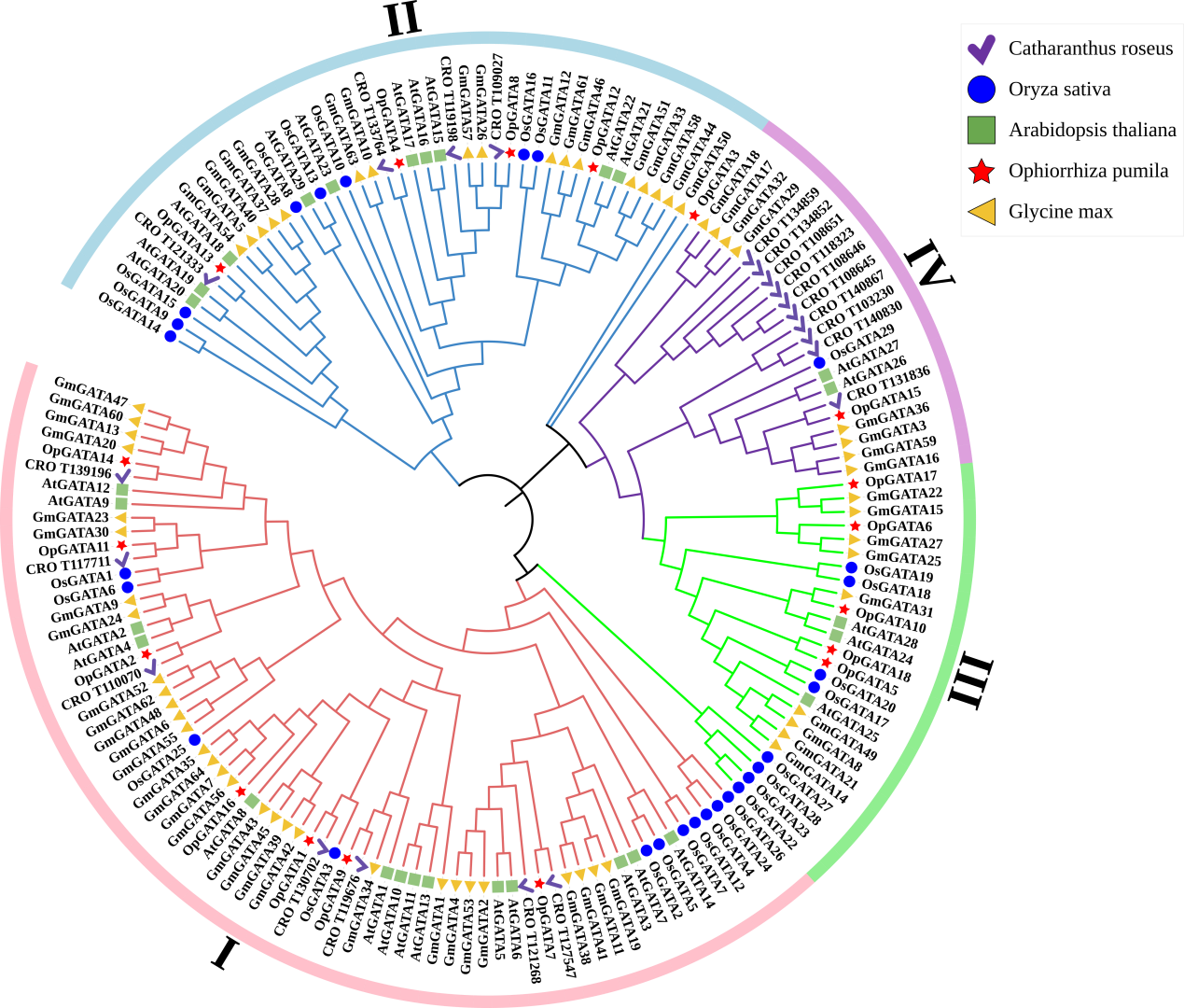


Figure S2 Analysis of all OpGATA promoter sequences.


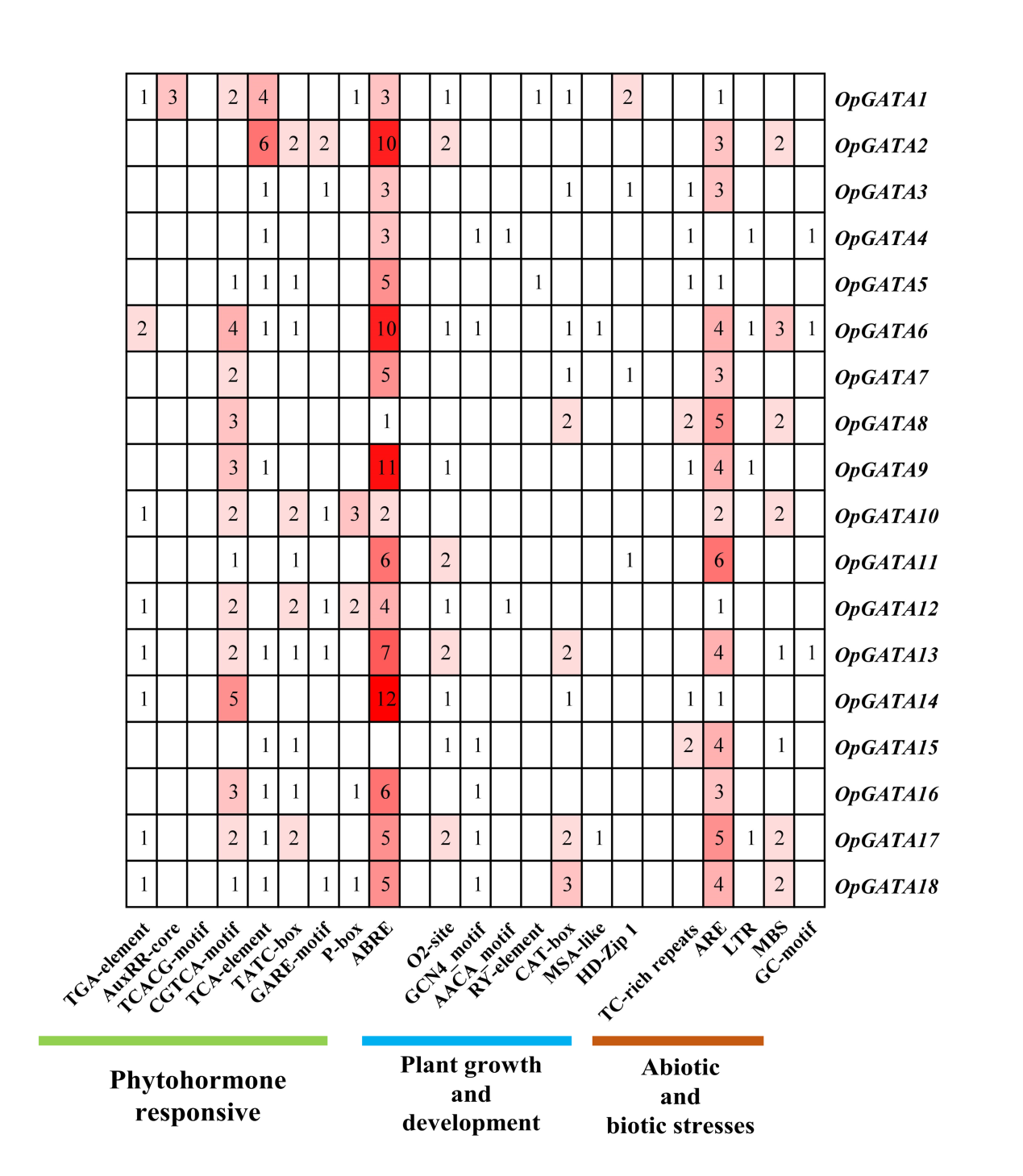

Supplement: Supplementary file 2 — Additional file 2. [file 12864_2022_8484_MOESM2_ESM.docx]
